# Editorial: Algae and microalgae-bacteria based technology for sustainable wastewater treatment

**DOI:** 10.3389/fmicb.2023.1263955

**Published:** 2023-08-08

**Authors:** Xiaoyuan Zhang, Zhongfang Lei, Shijian Ge, Bin Ji, Bing Zhang

**Affiliations:** ^1^Engineering Laboratory of Low-Carbon Unconventional Water Resources Utilization and Water Quality Assurance, College of Environmental Science and Engineering, Nankai University, Tianjin, China; ^2^Faculty of Life and Environmental Sciences, University of Tsukuba, Tsukuba, Japan; ^3^Jiangsu Key Laboratory of Chemical Pollution Control and Resources Reuse, School of Environmental and Biological Engineering, Nanjing University of Science and Technology, Nanjing, Jiangsu, China; ^4^Department of Water and Wastewater Engineering, School of Urban Construction, Wuhan University of Science and Technology, Wuhan, China; ^5^College of Environment and Ecology, Chongqing University, Chongqing, China

**Keywords:** algae, microalgae-bacteria, carbon capture, carbon neutrality, circular economy, resource recovery

With the fast-evolving global climate change, increasing effort has been devoted to the ways of mitigating greenhouse gases (GHGs) emissions from wastewater treatment plants (WWTPs). Therefore, great attention has been paid to carbon-neutral wastewater treatment technologies. As microalgae can grow upon CO_2_, algae and microalgae-bacteria consortia are believed to serve as a carbon sink for wastewater treatment, which have attracted increasing interest in recent years. Algae- and microalgae-bacteria based technologies have been widely explored for carbon-neutral wastewater treatment due to their unique feature of carbon capture and nutrients recovery.

The current Research Topic highlighting “*Algae and microalgae-bacteria based technology for sustainable wastewater treatment”* consists of five publications, which offer in-depth insights into the latest development of emerging algae and microalgae-bacteria based processes for various applications. In the article by Zhang et al., a revolving algae biofilm-based photosynthetic microbial fuel cell was reported for simultaneous energy recovery, pollutants removal and algae production from wastewater. In this system, both chemical oxygen demand (COD) and ammonium were efficiently removed with the maximum power density of 33.1 mW/m^2^ and a biomass production yield of 30 g/m^2^ being achieved. Their results show that revolving algae biofilm can be used as a bio-cathode in the photosynthetic microbial fuel cell for concurrent wastewater treatment and energy production.

Wang et al. tried a newly isolated microalgae *Desmodesmus* sp. SNN1 for the treatment of secondary effluent discharged from a WWTP. They found that about 53% of COD, 89% of total nitrogen and 95% of total phosphorus were removed in addition to 168.33 mg/L of lipids produced. It is expected that SNN1 growing well on the secondary effluent can more efficiently remove soluble nutrients.

On the other hand, microalgae biomass harvesting is technically challenging and economically costly. Lee et al. investigated algal mats that naturally agglomerated into a lump and float on water surfaces between filamentous algae (*Halomicronema* sp.) and extracellular polymeric substance (EPS)-producing algae (*Chlamydomonas* sp.). In this study, EPS produced by microalgae in certain environments were found to make up selected mats. By replicating the interactions between different types of algae, these biomimetic simulations provided insights into the conditions under which naturally robust clusters were formed and maintained for a long period of time without microbial detachment being observed. In addition, the new cultivation method proposed in this study could provide a good reference for the development of a sustainable and cost-effective technology to enhance the formation of a microalgal mat without the facilities or attachment carriers required for existing biofilm systems.

Excess discharge of phosphorus into natural water bodies has been identified as one of the main causes for eutrophication. Algae are able to uptake wastewater nutrients through microbial assimilation. Schaedig et al. reported a revolving algal biofilm (RAB) system as a polishing step for handling wastewater phosphorus prior to discharged into aquatic ecosystems. In this work, among 101 microalgae strains isolated from RAB systems, high levels of poly-P were found to be accumulated in multiple species of diatoms and one species of green algae. It is expected that the P-hyperaccumulating microalgae isolated in this study could potentially be further used to improve P removal and recovery from wastewater in other microalgal wastewater treatment systems (e.g., waste stabilization ponds or photobioreactors) and may also possess other valuable phenotypes for downstream applications of wastewater-grown algal biomass, such as high lipid content for biofuel production.

The circular economy is game-changing wastewater management with the aim to maximize water reuse, energy generation and resource recovery from wastewater. In the article by Sun et al., algal proteins were extracted from red algae *Eucheuma cottonii* (*E. cottonii*) and further hydrolyzed with proteolytic enzymes. Results show that the antioxidant peptides derived from *E. cottonii* can be used in a way like the natural antioxidants to treat chronic cardiovascular diseases caused by oxidative damage. Given such a context, algae and microalgae-bacteria can further offer a promising avenue to foster indirect energy and resource recovery from municipal wastewater in the forms of biomethane, biofertilizer, biodiesel, single-cell proteins, and natural pigments, etc.

To conclude, algae and microalgae-bacterial technologies may provide an engineering alternative for concurrently addressing the issues associated with wastewater reclamation and carbon emissions, meanwhile producing valuable biomaterials and bioenergy. It is expected that this Research Topic can help to trigger more R&D interests in developing microalgal and microalgal-bacterial technologies for maximizing the environmental and economic benefits of wastewater industry.

## Author contributions

XZ: Writing—original draft, Writing—review and editing. ZL: Writing—review and editing. SG: Writing—review and editing. BJ: Writing—review and editing. BZ: Writing—review and editing.

